# Outcome and Prognosis of Invasive Treatment for Hepatocellular Carcinoma in Very Elderly Patients Over 90 Years Old

**DOI:** 10.5152/tjg.2025.24163

**Published:** 2025-01-13

**Authors:** Keiji Yokoyama, Hiroaki Tokushige, Takahiro Nagata, Takashi Miyayama, Kumiko Shibata, Hiromi Fukuda, Ryo Yamauchi, Atsushi Fukunaga, Kazuhide Takata, Takashi Tanaka, Satoshi Shakado, Shotaro Sakisaka, Fumihito Hirai

**Affiliations:** Department of Gastroenterology and Medicine, Fukuoka University Faculty of Medicine, Fukuoka, Japan

**Keywords:** Hepatocellular carcinoma, percutaneous ethanol injection, radiofrequency ablation, transcatheter arterial chemoembolization, elderly

## Abstract

**Background/Aims:**

To evaluate invasive treatment outcomes for hepatocellular carcinoma (HCC) in patients aged over 90 years.

**Materials and methods:**

Twenty-six patients were included. Information on backgrounds, course of treatment, outcomes, and changes in Child–Pugh (CP) score and performance status (PS), as well as a comparison of treatment-related complications and 2-year survival after treatment, were retrospectively examined and compared with 311 patients aged under 90 years who were matched under the same conditions.

**Results:**

The mean patient age was 91.1 years. Seventeen patients had cirrhosis. The CP score was ≤ 7 across all cases. The Barcelona Clinic Liver Cancer stage was ≤B across all cases. The initial treatments were percutaneous local treatment and transcatheter arterial chemoembolization in 14 and 12 cases, respectively. Several patients with postoperative delirium and cognitive impairment were observed. No significant early post-treatment declines were observed in hepatic reserve and PS. The cumulative survival rates after treatment were 77.8% and 61.5% at 12 and 24 months, respectively. The 2-year survival after treatment for patients aged under 90 years was 87.4% and 75.7% at 12 and 24 months, respectively. No significant difference was observed in treatment-related complications or 2-year survival rates between patients aged over and under 90 years.

**Conclusion:**

This is the first report to analyze the course of invasive treatment for HCC in patients aged over 90 years. Safety was ensured if hepatic reserve and PS were maintained. The 2-year survival was comparable with that of patients aged under 90 years, suggesting benefit from HCC treatment.

**Main Points**

- This is the first report to analyze the course of invasive treatment for HCC in elderly patients aged over 90 years.
- No significant difference was observed in the treatment-related complications or 2-year survival rate after treatment between patients over and under 90 years old who were treated for the same condition.
- Very elderly patients with liver cirrhosis benefited from HCC treatment if the performance status and liver reserve were preserved.
- Whether invasive treatment truly contributed to prolonged prognosis remained unclear due to selection bias before treatment, warranting future comparisons with an untreated group and studies across multiple centers.

## Introduction

Hepatocellular carcinoma (HCC) is the sixth most commonly diagnosed cancer and the third most prevalent cause of cancer-related deaths worldwide. The high mortality rate associated with HCC is a major global health problem,^1^^,^^2^ The HCC-related mortality rate steadily increases with age, and the age at death increases.^3^ Moreover, an aging society worldwide and increased longevity in the future indicate that the number of elderly cancer patients is predicted to increase in the future.^4^ In Japan, the average age of patients with HCC has been increasing, and the proportions of elderly HCC patients and the adjusted HCC mortality have increased in recent years.^5^

The treatment of elderly patients with HCC is considerably more complicated than treating younger patients due to the presence of comorbidities such as cardiovascular and respiratory diseases, diabetes, renal dysfunction, and changes in drug metabolism.^6^ Consequently, many elderly patients often do not receive the best possible cancer treatment, as it is frequently withheld due to concerns about minimal survival benefits and potential toxicity.^7^^,^^8^

Current effective therapies for HCC, including surgical resection (SL), liver transplantation (LT), transcatheter arterial chemoembolization (TACE), hepatic arterial infusion chemotherapy, percutaneous radiofrequency ablation (RFA), percutaneous ethanol injection (PEIT), percutaneous microwave coagulation therapy (PMCT), molecular targeted agents (MTAs), immune checkpoint inhibitors (ICIs), as well as combination therapies with ICI and MTA, are available for use in clinical practice.^9^^-^^12^ Optimal treatment for HCC should be tailored to each patient, considering their performance status (PS), tumor characteristics, hepatic reserve, and comorbidities. However, current HCC management guidelines do not provide age-specific strategies.^13^^,^^14^

These tendencies have heightened the demand in Japan for research on the clinical features and treatment outcomes of elderly HCC patients. In the present study, we performed an analysis of retrospective data on invasive treatments for patients aged >90 years at our hospital and evaluated the outcomes and prognosis.

## Materials and Methods

**Figure 1.**
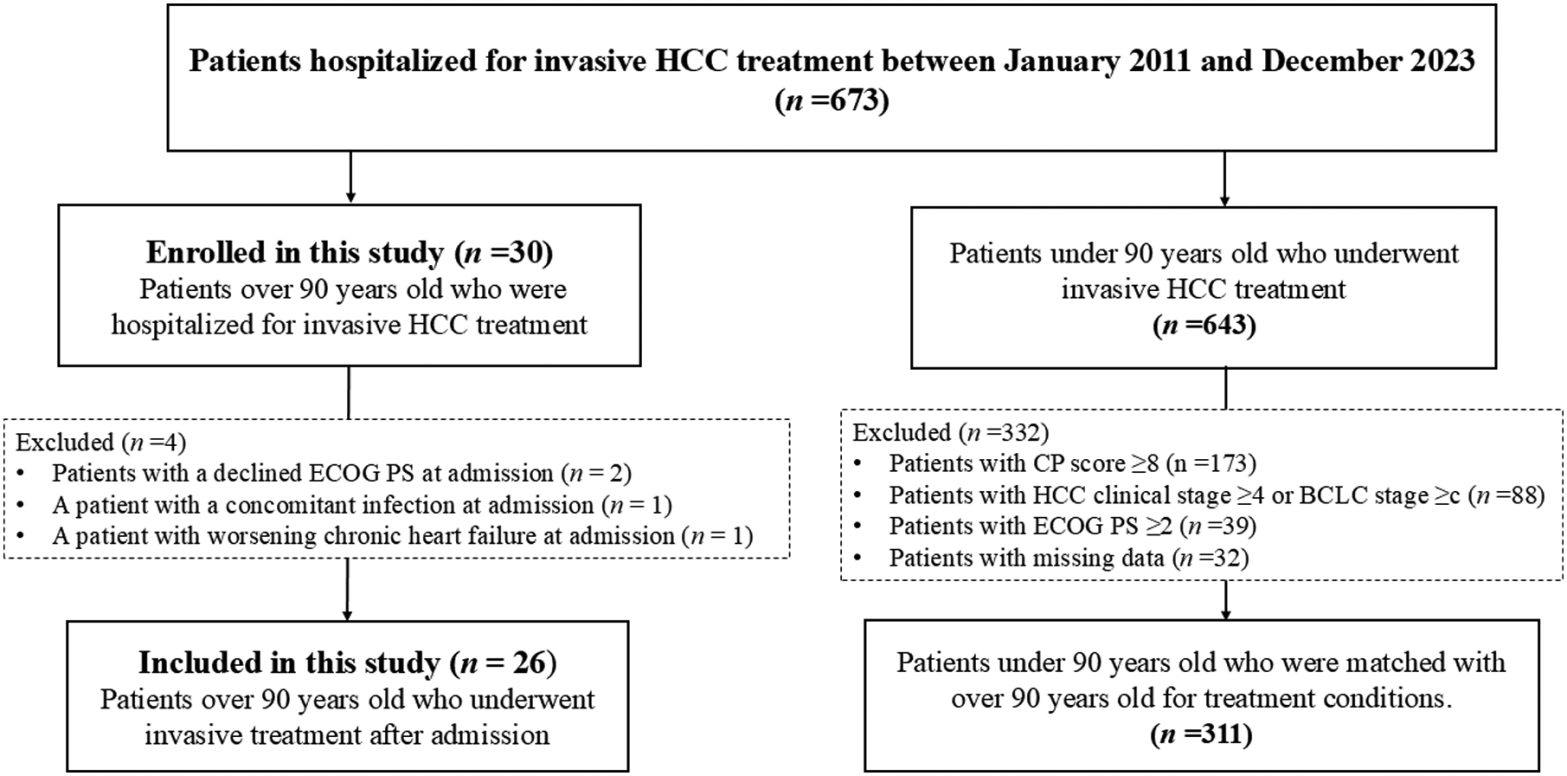
Inclusion criteria of the patients in this study and the selection of the control group comprising patients under 90 years old matched with those over 90 years old for treatment conditions. BCLC, Barcelona Clinic Liver Cancer; CP, Child–Pugh; ECOG, Eastern Cooperative Oncology Group; HCC, Hepatocellular carcinoma; PS, performance status.

In this study, we extracted the data of 673 patients hospitalized for invasive HCC at Fukuoka University Hospital between January 2011 and December 2023. Of these, we initially enrolled 30 patients aged over 90 years old; 4 patients were excluded as they were untreated, including 2 with a decline in PS at admission, 1 with a concurrent infection, and 1 with worsening heart failure. Overall, 26 patients who underwent invasive treatment (41 sessions) after admission were included in this study (Figure 1).

In all cases, after the benefits and risks of the treatment were fully understood, approval with intensive informed consent was obtained from the patients and their families. In this study, the following items were examined in detail: 1) patient background at the time of treatment, 2) treatment method and the number of treatments, 3) treatment-related complications, 4) cumulative survival and progression-free survival (PFS) after treatment, 5) cause of death in deceased patients, 6) changes in hepatic reserve after treatment, 7) changes in Eastern Cooperative Oncology Group (ECOG) PS, and 8) comparison of treatment-related complications and 2-year cumulative survival after treatment with 311 patients under 90 years old who were matched for HCC stage, hepatic reserve, and treatment conditions in the control group.

### Statistical Analysis

All statistical analyses were performed using JMP version 14.0 (SAS Institute, Cary, NC, USA), with *P *< .05 being considered statistically significant. Results are presented as the mean ± standard deviation (SD). Comparisons between groups were conducted using a *t*-test for differences in means and a chi-square test for differences in proportions. Survival curves were plotted using Kaplan–Meier curves. Changes in clinical data were calculated using the Wilcoxon signed-rank test. Differences between the 2 independent groups were analyzed using the log-rank test.

### Ethical Statement

The study protocol was approved by the Ethics Committee of Fukuoka University Hospital (approval number: H22-01-004, date: January 14, 2022). This study was conducted in compliance with the principles of the Declaration of Helsinki and the Ethical Guidelines for Medical Research of the Ministry of Health, Labor, and Welfare. The collected data were anonymized. Owing to the retrospective nature of the study, informed consent and the opportunity to opt out were waived by the Ethics Committee. This study was included in the list of studies approved by the Ethics Committee of Fukuoka University Hospital (http://www.med.fukuoka-u.ac.jp/research/life_med_ethic/).

## Results

### Patient Background at the Time of Treatment

The characteristics of the 26 patients are listed in Table 1. The mean age of the patients was 91.1 ± 1.27 years and the sex ratio (male/female) was 15/11. Fifteen patients were over 90 years old at the time of first treatment. The etiology of liver disease was viral hepatitis, metabolic dysfunction associated steatohepatitis (MASH), alcohol consumption, primary biliary cholangitis, hepatitis B core (HBc) antibody positive, and others in 14, 5, 1, 1, 1, and 4 (2 patients with type 2 diabetes, 2 patients with cryptogenic) patients, respectively. There were 17 and 9 patients with and without cirrhosis, respectively, with a mean Child–Pugh (CP) score of 5.67 ± 0.82. Child–Pugh score was less than 7 in all cirrhosis patients. The mean model for end-stage liver disease (MELD) score was 8.69 ± 0.36 ng/mL, the clinical stage of HCC was I, II, III, and IV in 7, 13, 6, and 0 patients, respectively, while the Barcelona Clinic Liver Cancer (BCLC) stage was 0, A, B, and C in 7, 13, 6, and 0 patients, respectively. The mean alpha-fetoprotein (AFP) value was 637.4 ± 316.5 ng/mL, tumor maximum diameter was 2.73 ± 0.27 cm, and tumor number was 2.19 ± 0.40.

### Treatment Method and the Number of Treatments

The treatment method, including RFA, TACE, and PEIT, was used as the initial treatment in 13, 12, and 1 patient(s), respectively, while RFA, TACE, PEIT, or PMCT was used for all 41 sessions in 23, 16, 1, and 1 patient(s), respectively (Table 2). Out of the 13 patients who underwent initial RFA, 10 patients (76.9%) achieved complete ablation without recurrence for at least 6 months post-RFA. Transcatheter arterial chemoembolization was a localized treatment targeting the nutrient vessels of the tumor in all cases. The number of treatments for patients over 90 years old was 1, 2, and 3 times or more in 17, 6, and 3 patients, respectively. Most cases were treated once and followed up; however, some cases were treated repeatedly.

### Treatment-Related Complications

A list of complications associated with the treatment across 41 sessions is shown in Table 3. Fever was the most common complication that occurred in 24 sessions (58.5%), followed by abdominal pain or pain in the treatment area in 16 (39.0%), and nausea in 6 (14.6%) sessions. Postoperative delirium was observed in 2 sessions (4.9%) and mild cognitive impairment in 1 session (2.4%). One session (2.4%) recorded intra-abdominal bleeding after RFA; however, no abnormalities in vital signs were observed in the patient after transfusion of 2 units of concentrated red blood cells, and the intra-abdominal bleeding resolved spontaneously. All patients were discharged without serious complications. There were no cases of a significant decline in hepatic reserve or ECOG PS in the early stage (within 1 month) after treatment and after discharge, and no deaths were reported.

### Cumulative Survival and Progression-Free Survival After Treatment

**Figure 2.**
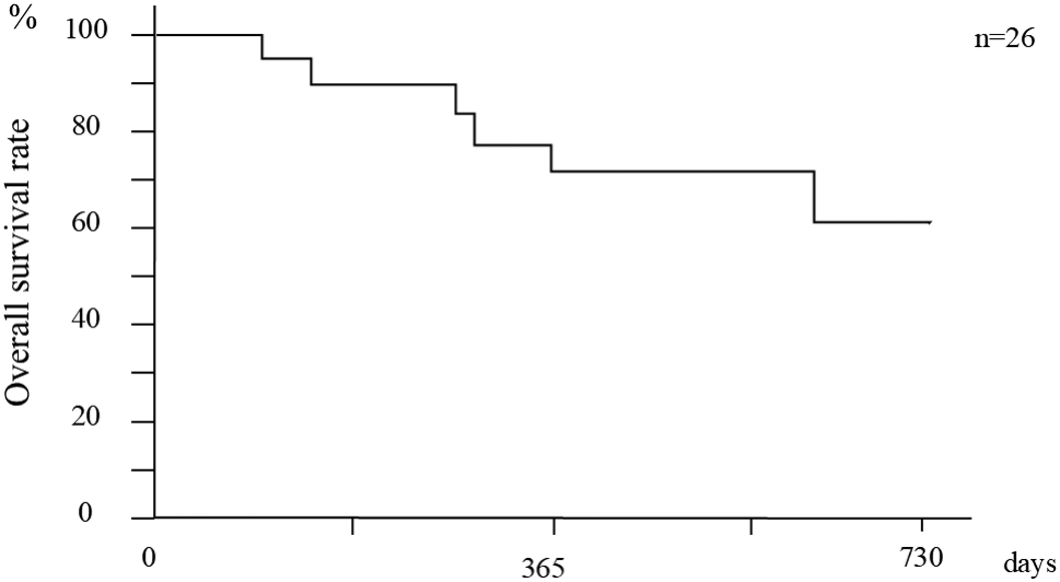
Overall cumulative survival rates.

**Figure 3.**
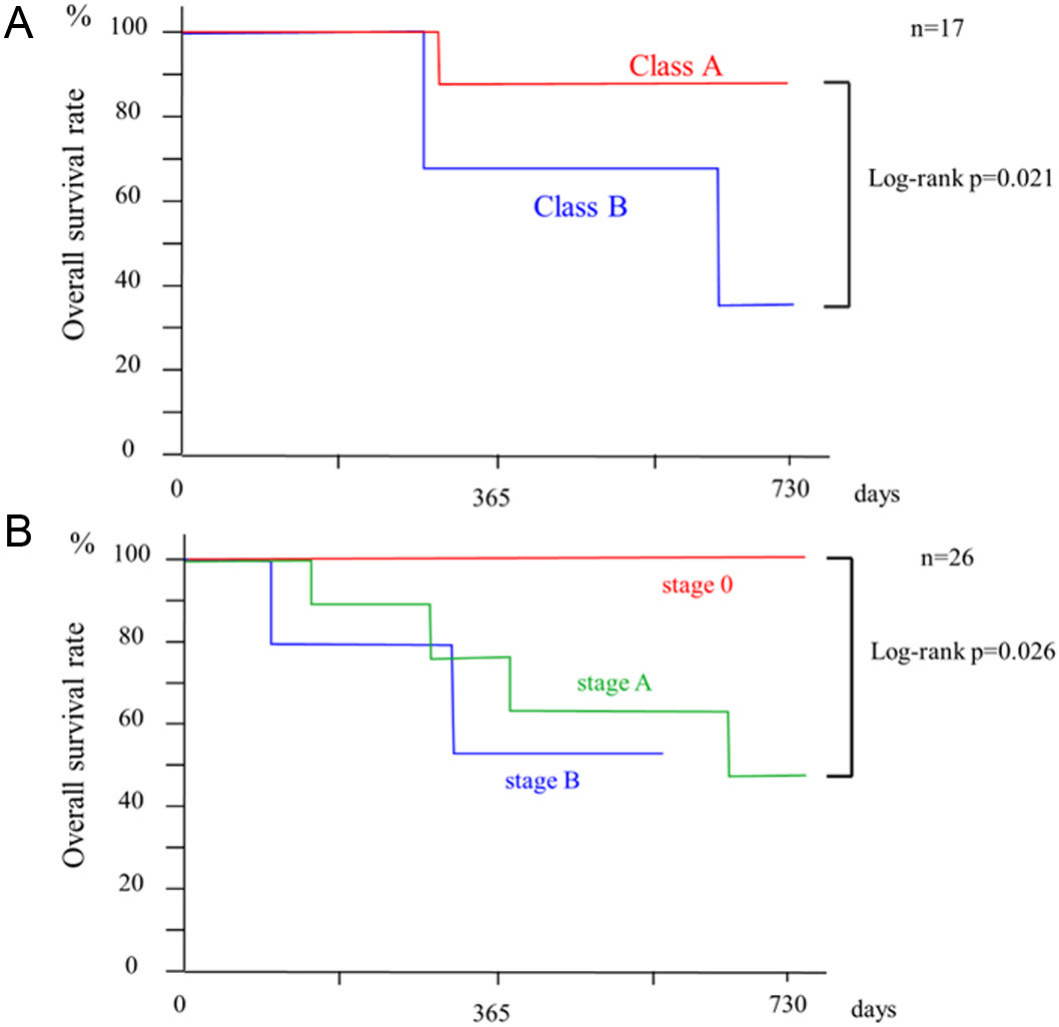
A. Cumulative survival of different CP classes in 17 patients with liver cirrhosis. B. Cumulative survival according to BCLC stage. BCLC, Barcelona Clinic Liver Cancer; CP, Child–Pugh; n, number.

The cumulative overall survival (OS) rate after initial treatment (median observation period: 382 days) was 89.7% at 6 months, 77.8% at 12 months, 71.8% at 18 months, and 61.5% at 24 months (Figure 2). In 17 patients with liver cirrhosis, the OS at 6, 12, 18, and 24 months for CP class A was 100%, 87.5%, 87.5%, and 87.5%, respectively, while that for CP class B was 100%, 66.7%, 66.7%, and 33.3%, respectively. The OS was significantly higher for CP class A than for CP class B (*P *= .021) (Figure 3A).

**Figure 4.**
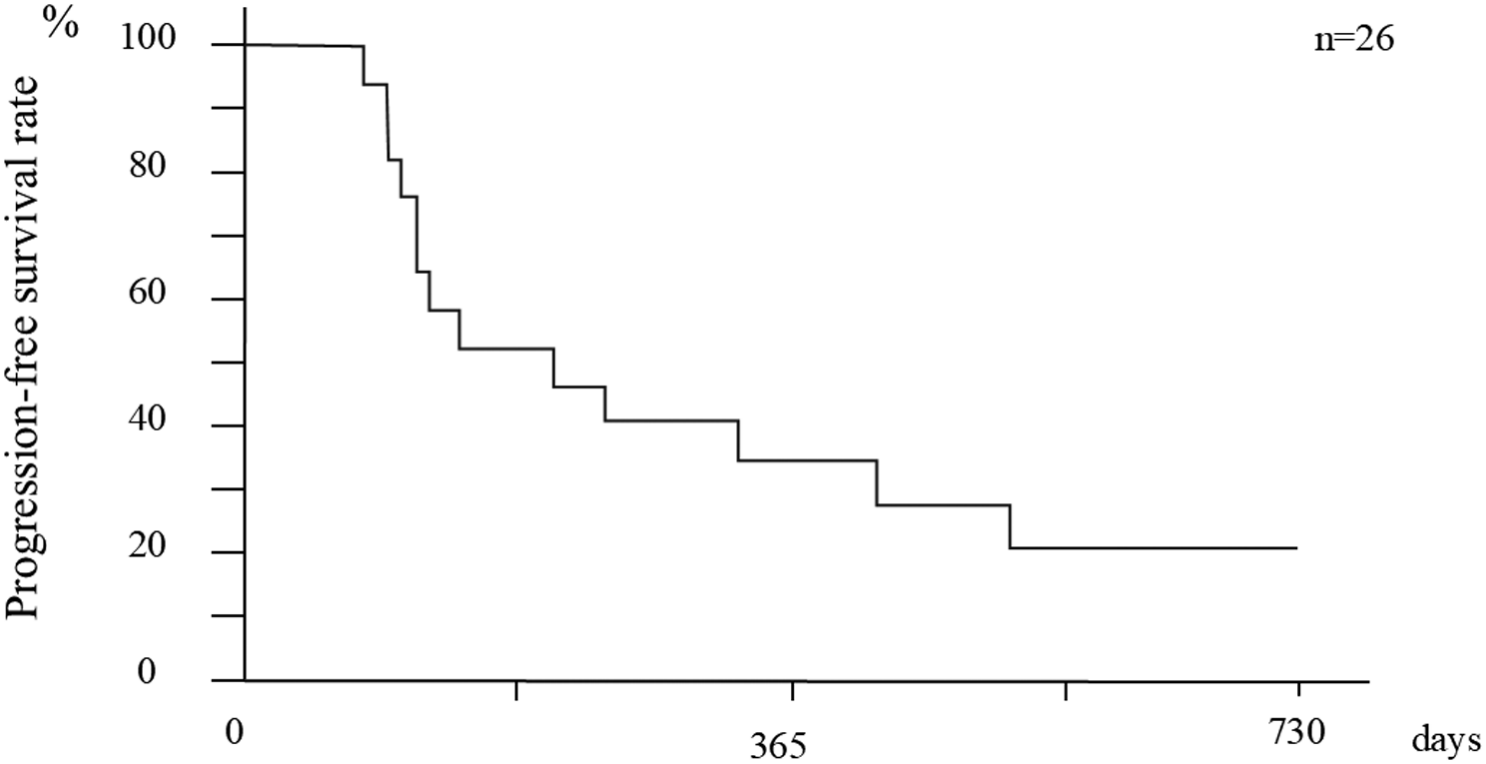
Overall progression-free survival rates.

Regarding the BCLC stage, the cumulative OS of stage 0 was 100% over the entire observation period, while that at 6, 12, 18, and 24 months of stage A as well as stage B was 88.9%, 76.2%, 63.5%, and 47.6%, as well as 80.0%, 53.3%, 53.3%, and not reached, respectively. The OS of BCLC stage 0 was significantly longer than that of stage A (*P *= .026) (Figure 3B). The overall PFS after initial treatment (median observation period, 213 days) at 6, 12, 18, and 24 months was 52.9%, 35.3%, 21.2%, and 21.2%, respectively (Figure 4).

### Cause of Death Among the Deceased Patients

Of the 9 patients who died, 3 were due to HCC, and the remaining 6 were due to infection (4 patients), aortic rupture (1 patient), and senility (1 patient). Deaths due to HCC accounted for 33.3% of all deaths.

### Changes in Hepatic Reserve After Treatment

**Figure 5.**
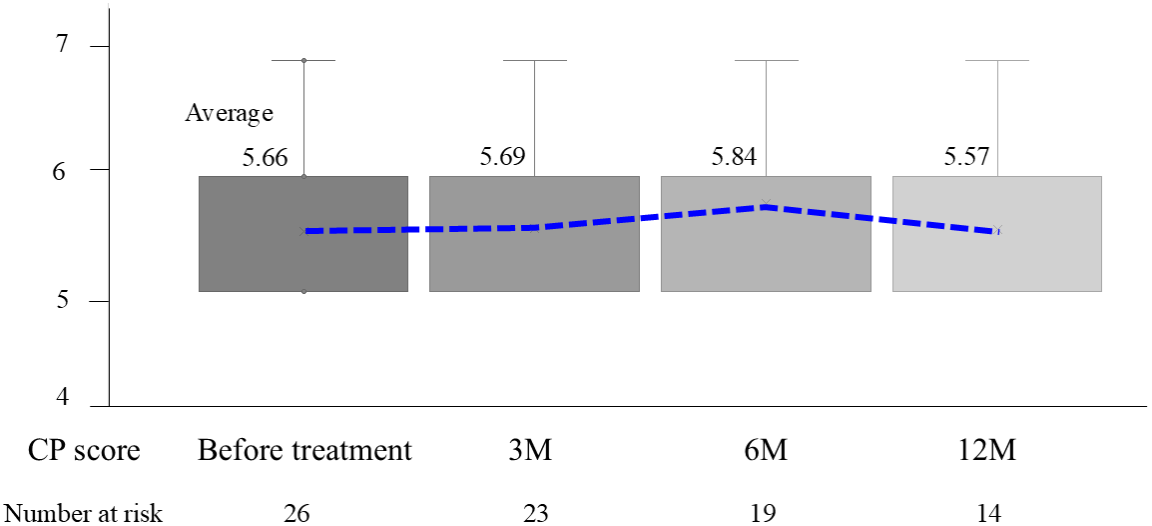
Changes in CP score. CP, Child–Pugh; M, month.

**Figure 6.**
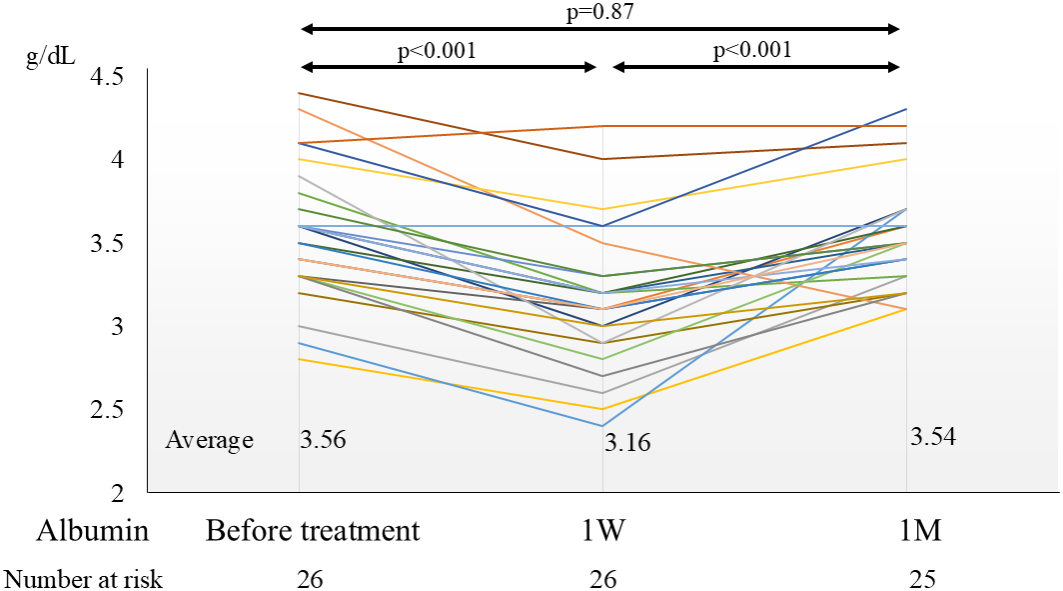
Changes in albumin values. M, month; W, week.

The mean CP score before treatment and at 3, 6, and 12 months after treatment (non-cirrhotic patients were converted into a CP score of 5) was 5.66, 5.69, 5.84, and 5.57, respectively (Figure 5). There were no cases of significantly decreased hepatic reserve. The mean albumin level (g/dL) before treatment and after 1 week and 1 month of treatment was 3.56, 3.16, and 3.54, respectively (Figure 6). Although the nutritional status of the patients deteriorated during hospitalization, it recovered quickly after discharge.

### Changes in Eastern Cooperative Oncology Group Performance Status

The ECOG PS (0/1) before treatment, as well as after 3, 6, and 12 months of treatment, was 21/5, 18/5, 15/4, and 11/2, respectively. No patient showed a significant decrease in PS within a short period due to treatment (Table 4).

### Comparison of Treatment-Related Complications and 2-Year Cumulative Survival After Treatment Between Patients Over and Under 90 Years Old

A total of 311 patients aged under 90 years were included in the control group, matched for HCC stage, hepatic reserve, and treatment conditions as those of the group. The exclusion from this group comprised 173 patients with a CP score of ≥8, 88 patients with HCC clinical stage IV or BCLC stage C, 39 patients with an ECOG PS of ≥2, and 32 patients with missing data (Figure 1).

Table 5 shows the comparison of patient backgrounds at the time of treatment between patients over and under 90 years old. Compared to the treatment group comprising patients over 90 years old, those who were aged under 90 years exhibited no significant differences in sex ratio, cause of liver disease, proportion of patients with cirrhosis, mean CP score, and MELD score for cirrhosis, clinical stage of HCC, BCLC stage, AFP value, tumor maximum diameter, tumor number, and treatment methods.

The comparison of treatment-related complications is shown in Table 6. No significant differences in complications were observed when comparing the treatment group of patients over 90 years old and those under 90 years old.

**Figure 7.**
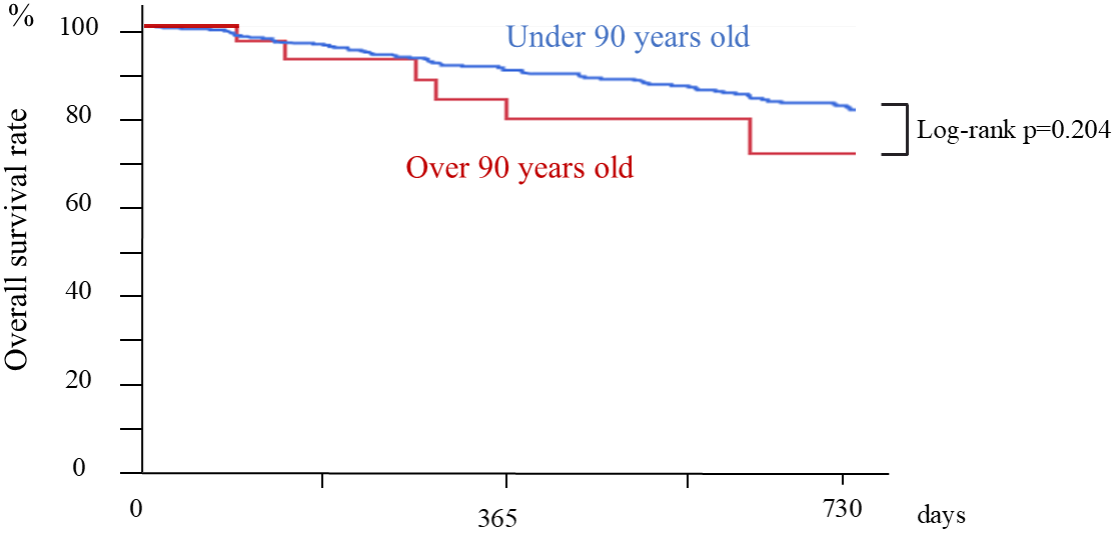
Cumulative survival according to patients over and under 90 years old.

The 2-year cumulative survival after initial treatment (median observation period: 1528 days) of patients under 90 years old was 94.1% at 6 months, 87.4% at 12 months, 82.0% at 18 months, and 75.7% at 24 months (Figure 7). No significant difference was observed for the 2-year survival after treatment between patients over and under 90 years old (*P *= .204).

## Discussion

Patients over 90 years old who were treated at our hospital exhibited a preserved ECOG PS. Notably, even those with cirrhosis had a relatively good hepatic reserve with a CP score of ≤7. In addition, invasive treatment, such as TACE and RFA, was localized in all cases, and no serious complications were observed. The safety of invasive treatment was ensured in patients who fulfilled the criteria.

Transcatheter arterial chemoembolization is a commonly used non-surgical treatment for elderly patients with HCC, showing effectiveness in extending survival.^7^^,^^15^^,^^16^ A prospective study involving 102 patients across 3 age groups (>75 years, 65-75 years, and <65 years) performed with TACE for unresectable HCC, based on the European Association for the Study of the Liver (EASL) criteria, found no differences in survival and incidence of complications among the groups. This suggests that older age does not correlate with higher complication rates.^17^ Within a substantial group of 1040 patients with HCC performed with TACE, only one study reported a significant difference in survival between patients aged younger and older than 70 years, and TACE-related mortality was not significantly different between the 2 groups (*P *= .49), concluding that the efficacy and tolerability of TACE are similar for elderly and younger patients with HCC.^18^

Surgical resection, LT, PEIT, PMCT, and RFA are treatments for early-stage HCC. In particular, RFA avoids the complications arising from general anesthesia and is less invasive, with decreased perioperative risks and less harm to hepatic reserve, which could be advantageous for elderly patients with a higher risk profile, making it an increasingly favored treatment selection for elderly patients with HCC.^19^ The American Association for the Study of Liver Diseases and EASL guidelines recommend RFA as a treatment option for patients with compensated cirrhosis whose HCCs are <5 cm.^20^^,21^ Numerous studies have highlighted the benefits of RFA for elderly people aged around 70 years.^22^^,^^23^ In a report of RFA mortality and complication rates within a substantial group of 54 145 HCC patients from a national database in Japan, it was found that age was significantly linked to in-hospital mortality for patients aged >70 years.^24^

Regarding systemic chemotherapy, which was not administered in this study, tyrosine kinase inhibitors such as sorafenib have been reported to achieve similar PFS and OS in elderly and young patients with advanced HCC.^25^ However, morbidities, such as neutropenia, malaise, and mucositis, occur more frequently in older patients.^26^ Immune checkpoint inhibitors may be a potential non-surgical treatment option for elderly patients with HCC; however, further studies are required.^27^

Based on these studies, although many showed no significant difference in survival time and complications after treatment, most of the cutoff values for elderly and non-elderly patients were between 65 and 75 years. No study has specifically focused on very elderly patients aged over 90 years, as in this study. To the best of our knowledge, this is the first preliminary report to analyze the course of invasive treatment for HCC in elderly patients aged over 90 years.

In this study, HCC-related deaths accounted for 33% of all deaths. Since a large proportion of deaths were due to other diseases, obtaining the true prognosis of HCC treatment proves difficult, which is an issue for future studies. In addition, the low PFS may be due to a larger proportion of patients being treated for disease control rather than for curative treatment.

In this study, delirium, cognitive decline, and poor nutritional status have been observed in some patients due to hospitalization and invasive treatments. Frailty is known to increase to 10% in patients aged >65 years and 25%-50% in those >85 years of age, as well as significantly with cancer stress and chemotherapy, which should be prevented through rehabilitation during hospitalization, appropriate nutritional management, and other multidisciplinary efforts.^28^^-^^30^

This study has limitations, particularly regarding whether invasive treatment truly contributed to the prolonged prognosis, considering the selection bias of cases. However, the survival rate 2 years after treatment was comparable to that of cases under 90 years old, considering factors such as good PS before treatment and the small number of deaths from liver cancer. Future research should address these limitations by comparing outcomes with an untreated group and conducting studies across multiple centers.

This is the first preliminary report to analyze the course of invasive treatment for HCC in elderly patients aged over 90 years. Overall, very elderly patients benefited from HCC treatment. Invasive treatment for HCC showed assured safety in patients aged over 90 years and was not precluded in patients with retained PS and relatively good hepatic reserve with CP scores of 7 or less, even in cirrhosis. Furthermore, the 2-year survival rate after treatment was comparable to that of cases under 90 years old, which is markedly important data for evaluating the benefits of HCC treatment. However, more clinical data are needed to determine the selection criteria to maximize treatment efficacy, especially in very elderly patients with HCC whose clinical condition, cancer stage, and comorbidities must be carefully evaluated to ensure therapeutic efficacy. Since it is not clear whether invasive treatment for HCC contributes to prolonged life expectancy, the current realistic treatment strategy would be for patients to be evaluated individually and treated flexibly to determine treatment options using the results of this study as a basis.

## Figures and Tables

**Table 1.** Patient Background at the Time of Treatment

Number of patients	**26**
Age (in years, expressed as mean ± SD)	91.1 ± 1.27
Sex (male/female)	15/11
Etiology	
Viral hepatitis	14 (53.8%)
MASH	5 (19.2%)
Alcohol consumption	1 (3.8%)
Primary biliary cholangitis	1 (3.8%)
HBc-Ab positive	1 (3.8%)
Others	4 (15.4%)
History of HCC treatment at less than 90 years of age	
Presence	11 (42.3%)
Absence	15 (57.7%)
Liver cirrhosis	
Presence	17 (65.4%)
Absence	9 (34.6%)
Child–Pugh classification in liver cirrhosis	
Class A; score 5 (n = 10), score 6 (n = 4)	14 (82.4%)
Class B; score 7 (n = 3), score 8-9 (n = 0)	3 (17.6%)
Class C	0 (0.0%)
Child–Pugh score in liver cirrhosis (mean ± SD)	5.67 ± 0.82
MELD score (mean ± SD)	8.69 ± 0.36
ECOG PS	
0	21 (80.8%)
1	5 (19.2%)
HCC clinical stage	
I	7 (26.9%)
II	13 (50.0%)
III	6 (23.1%)
IV	0 (0.0%)
BCLC stage	
0	7 (26.9%)
A	13 (50.0%)
B	6 (23.1%)
C	0 (0.0%)
AFP value (mean ± SD)	637.4 ± 316.5
Tumor maximum diameter (mean ± SD)	2.73 ± 0.27
Tumor number (mean ± SD)	2.19 ± 0.40

AFP, alpha-fetoprotein; BCLC, Barcelona Clinic Liver Cancer; ECOG, Eastern Cooperative Oncology Group; HCC, hepatocellular carcinoma; MASH, metabolic dysfunction associated steatohepatitis; MELD, model for end-stage liver disease; n, number; PS, performance status; SD, standard deviation.

**Table 2.** Treatment Methods in 26 Patients at 41 Sessions

Treatment Methods	Initial Treatment (n = 26)	All Sessions (n = 41)
RFA	13	23
TACE	12	16
PEIT	1	1
PMCT	0	1

n, number; PEIT, percutaneous ethanol injection; PMCT, percutaneous microwave coagulation therapy; RFA, radiofrequency ablation; TACE, transcatheter arterial chemoembolization.

**Table 3.** Complications Due to Treatment in 41 Sessions

Complication	Number of Cases (%)
Fever	24 (58.5)
Abdominal pain	16 (39.0)
Nausea	6 (14.6)
Delirium	2 (4.9)
Drug eruption	1 (2.4)
Mild cognitive impairment	1 (2.4)
Vertigo	1 (2.4)
Temporary decrease in oxygenation	1 (2.4)
Urinary retention	1 (2.4)
Intra-abdominal bleeding after RFA	1 (2.4)

RFA, radiofrequency ablation.

**Table 4.** Changes in ECOG PS

ECOG PS	Before Treatment (n = 26)	After 3 M (n = 23)	After 6 M (n = 19)	After 12 M (n = 14)
0	21	18	15	12
1	5	5	4	2

ECOG, Eastern Cooperative Oncology Group; M, month n, number; PS, performance status.

**Table 5.** Comparison of Patient Backgrounds at the Time of Treatment Between Patients Over and Under 90 Years Old

	Over 90 Years Old	Under 90 Years Old	*P*
Number of patients	**26**	**311**	
Age (in years, expressed as mean ± SD)	91.1 ± 1.27	72.85 ± 0.51	<.0001
Sex (male/female)	15/11	213/98	.258
Etiology			
Viral hepatitis/MASH/alcohol consumption/others	14/5/1/6	185/29/57/40	.0652
Liver cirrhosis			
Presence/absence	17/9	250/61	.701
Child–Pugh classification in liver cirrhosis			
Class A/class B/class C	14/3/0	249/62/0	.575
Child–Pugh score in liver cirrhosis (mean ± SD)	5.67 ± 0.82	5.71 ± 0.04	.294
MELD score (mean ± SD)	8.69 ± 0.36	8.82 ± 0.11	.731
ECOG PS			
0/1/2	21/5/0	257/52/2	.875
HCC clinical stage			
I/II/III/IV	7/13/6/0	58/189/64/0	.499
BCLC stage			
0/A/B/C	7/13/6/0	57/186/68/0	.652
AFP value	637.4 ± 316.5	4113 ± 1427	.499
Tumor maximum diameter (mean ± SD)	2.37 ± 0.24	2.51 ± 0.07	.403
Tumor number (mean ± SD)	2.19 ± 0.40	3.21 ± 0.40	.142
Treatment methods			
RFA/TACE/PEIT/PMCT	13/12/1/0	131/165/13/2	.863

BCLC, Barcelona Clinic Liver Cancer; ECOG, Eastern Cooperative Oncology Group; HCC, hepatocellular carcinoma; n, number; MASH, metabolic dysfunction associated steatohepatitis; PEIT, percutaneous ethanol injection; PMCT, percutaneous microwave coagulation therapy; PS, performance status; RFA, radiofrequency ablation; SD, standard deviation; TACE, transcatheter arterial chemoembolization.

**Table 6.** Comparison of Treatment-Related Complications Between Patients Over and Under 90 Years Old

	Over 90 Years Old n = 41	Under 90 Years Old n = 311	*P*
Complication	n (%)	n (%)	
Fever	24 (58.5)	192 (61.7)	.692
Abdominal pain	16 (39.0)	139 (44.7)	.492
Nausea	6 (14.6)	56 (18.0)	.594
Delirium	2 (4.9)	11 (3.5)	.668
Drug eruption	1 (2.4)	8 (2.5)	.959
Mild cognitive impairment	1 (2.4)	4 (1.3)	.557
Vertigo	1 (2.4)	4 (1.3)	.557
Temporary decrease in oxygenation	1 (2.4)	7 (2.3)	.939
Urinary retention	1 (2.4)	2 (0.6)	.239
Intra-abdominal and/or pleural bleeding after RFA	1 (2.4)	12 (3.8)	.650

n, number; RFA, radiofrequency ablation.

## Data Availability

The data that support the findings of the study are available on request from the corresponding author.
